# Concomitant improvement in anti-saccade success rate and postural instability gait difficulty after rTMS treatment for Parkinson’s disease

**DOI:** 10.1038/s41598-021-81795-3

**Published:** 2021-01-28

**Authors:** Ken-ichi Okada, Mizuki Takahira, Tomoo Mano, Taichi Uga, Kuni Konaka, Koichi Hosomi, Youichi Saitoh

**Affiliations:** 1 Graduate School of Frontier Biosciences, Osaka University, 1-3 Yamadaoka, Suita, 565-0871 Japan; 2 Center for Information and Neural Networks (CiNet), National Institute of Information and Communications Technology, and Osaka University, 1-4 Yamadaoka, Suita, 565-0871 Japan; 3 Department of Neuromodulation and Neurosurgery, Osaka University Graduate School of Medicine, 2-2 Yamadaoka, Suita, 565-0871 Japan; 4 Department of Physical Therapy, Faculty of Health Science, Osaka Yukioka College of Health Science, 1-1-41 Soujiji, Ibaraki, 567-0801 Japan; 5 Department of Neurosurgery, Osaka University Graduate School of Medicine, 2-2 Yamadaoka, Suita, 565-0871 Japan; 6 Present Address: Department of Physiology, Hokkaido University School of Medicine, Sapporo, 060-8638 Japan; 7 Present Address: Department of Neurology, Nara Medical University, 840 Shijo-Cho, Kashihara, 634-8521 Japan

**Keywords:** Parkinson's disease, Parkinson's disease, Saccades

## Abstract

Parkinson’s disease (PD) is a progressive neurological disorder characterised by motor and non-motor deficits. Repetitive transcranial magnetic stimulation (rTMS) over the bilateral primary motor cortex at a high frequency (5 Hz or higher) is reported to be a potential treatment of PD. We aimed to assess the effect of rTMS on eye movement control in patients with PD in their ‘on’ state. We enrolled 14 patients with PD and assessed motor symptoms (Movement Disorder Society-Sponsored Unified Parkinson’s Disease Rating Scale; MDS-UPDRS) and eye movement performances (visually guided saccades, volitional anti-saccades, and small involuntary saccades during fixation) at baseline and after administering bilateral 10 Hz rTMS on leg region of the motor cortex. We confirmed that rTMS improved the MDS-UPDRS motor scores and found that rTMS improved the anti-saccade success rate, which requires adequate inhibition of the reflexive response. The improvement in anti-saccade success rate was correlated with that of the postural instability gait difficulty (PIGD) sub-scores of MDS-UPDRS and lower baseline Japanese version of the Montreal Cognitive Assessment scores. This result is consistent with previous findings that PIGD and inhibitory control deficits share common brain dysfunctions in PD. rTMS may alleviate dysfunctions of that circuit and have a clinical effect.

## Introduction

Parkinson’s disease (PD) is a progressive neurological disorder caused by dopaminergic neuron loss in the midbrain^1^. Tremor, rigidity, and bradykinesia are the typical PD motor symptoms, and many patients with PD also show non-motor deficits, including cognitive deficits^2^. Different PD subtypes could exist, and patients are initially categorised into either the tremor dominant or postural instability and gait difficulty (PIGD) subtypes^3,4^. Although symptom progression in PD varies widely, a majority of patients with PD experience PIGD in the advanced stage^3,4^. Previous studies have reported a correlation of PIGD with more severe motor and cognitive deficits^5^.

In addition to the aforementioned typical motor and non-motor deficits, several studies have reported eye movement disorders in patients with PD^6^. Previous studies have reported slowed latency and worsened accuracy of visually guided saccades in patients with PD with disease progression^7^ and with motor and cognitive impairment^8^. Furthermore, several studies have reported poor performances in the anti-saccade task^9–12^ that requires individuals to inhibit reflexive responses to a peripheral target and make voluntary saccades towards the opposite side. Specifically, patients with freezing of gait show significantly worse anti-saccade performances, indicating mutually impaired inhibitory control for gait and anti-saccade^13,14^. Saccades are the most studied eye movements and are controlled by various brain regions, with different patterns of saccade impairment reflecting pathologies in corresponding brain regions^15^. Currently, eye movement examination is considered as a useful tool for evaluating the clinical implications of the underlying pathophysiology and treatment effect^15–17^.

L-dopa application is the standard treatment for PD and is known to alleviate major motor symptoms, including bradykinesia and tremor. However, an increased duration of use reduces its efficacy and results in complications, including dyskinesia and psychological symptoms. Moreover, its therapeutic effect on PIGD is limited^18^, and that on eye movements remains controversial^19^. Non-pharmacological treatments for PD, including deep brain stimulation (DBS) targeting the subthalamic nucleus (STN)^20^, internal segment of the globus pallidus (GPi)^21^, ventral intermediate thalamic nucleus^22^, and pedunculopontine tegmental nucleus (PPN)^23^, have been recently developed. DBS of the GPi and STN improves locomotor activity^24^; however, it has varying effects on anti-saccade performance, which could be reflective of different therapeutic mechanisms^25^.

DBS has been established as a therapeutic option upon pharmacological treatment failure; however, it has limited availability and bears a risk of surgical complications. Recent reports show that repetitive transcranial magnetic stimulation (rTMS), a non-pharmacological and non-surgical approach, improves motor function in patients with PD. High frequency (5 Hz or higher) stimulation, targeting the primary motor cortex (M1), has been reported to have a significant effect on PD motor symptoms^26–29^. Specifically, gait performance has been improved by rTMS over the leg region of the motor cortex^30–33^. rTMS has been shown to induce changes in cortical excitability^28^. Moreover, it has been shown to affect basal ganglia circuits that are distant from the stimulation site, potentially underlying its therapeutic effect^34,35^. However, no studies report the effects of rTMS on eye movement control in patients with PD. Further, elucidating the effects of rTMS on saccades could provide clues regarding the mechanisms underlying the therapeutic effect of rTMS.

We aimed to assess the motor symptoms and saccade performance in patients with PD with and without rTMS treatment. Specifically, the effects of rTMS treatment on tremor and PIGD were evaluated using the Movement Disorder Society-Sponsored Revision of the Unified Parkinson’s Disease Rating Scale (MDS-UPDRS) sub-scores^36,37^. In addition, the effects of rTMS treatment on saccade performance were assessed by analysing the speed and accuracy of saccades based on visually guided saccade tasks, inhibitory control in the anti-saccade tasks, and small involuntary saccades during fixation.

## Results

### Effects of rTMS on MDS-UPDRS score and saccade performance

We examined the baseline and post-rTMS MDS-UPDRS Part 3 scores and saccade performance of 14 patients with PD. Table 1 summarises the background demographic and clinical characteristics for our study participants with PD.

**Table 1:** Demographics and clinical measures.

No.	Sex	Age (years)	Disease duration (month)	LEDD (mg)	MoCA-J	MDS-UPDRS					Tremor score	PIGD score	iAnti
						Total	Part 1	Part 2	Part 3	Part 4			
1	M	41	144	200	21	47	11	12	24	0	0.91	1.20	0.52
2	F	69	60	150	24	29	7	5	17	0	0.82	0.60	0.43
3	F	68	84	532	22	64	17	11	34	2	0.18	0.80	0.34
4	F	65	60	100	23	41	5	11	25	0	0.64	1.00	0.29
5	M	87	14	200	26	33	6	5	22	0	0.91	1.00	0.22
6	F	71	29	245	28	49	11	7	31	0	0.45	2.40	0.18
7	M	71	24	352	24	60	16	10	31	3	0.82	2.20	0.06
8	M	69	48	300	24	39	10	10	19	0	0.27	2.00	0.04
9	M	82	48	437	25	56	15	8	31	2	0.36	1.20	0.01
10	F	67	9	150	28	25	1	3	20	1	1.27	0.20	− 0.002
11	F	68	36	770	22	35	11	5	17	2	0.55	1.40	− 0.01
12	F	77	72	100	25	27	9	3	15	0	0.36	0.60	− 0.04
13	F	70	18	222	27	37	5	5	27	0	1.27	1.40	− 0.09
14	M	69	14^4^	1015	25	102	21	20	48	3	2.73	1.60	− 0.19
Mean ± SD	M : F = 6 : 8	69.6 ± 10.3	56.4 ± 43.2	340.9 ± 268.6	24.6 ± 2.2	46.0 ± 20.2	10.4 ± 5.5	8.2 ± 4.6	25.8 ± 8.8	0.9 ± 1.3	0.8 ± 0.6	1.3 ± 0.6	0.13 ± 0.21

*LEDD* levodopa equivalent daily dose, *MoCA-J* Japanese version of the Montreal Cognitive Assessment, *MDS-UPDRS* Movement Disorder Society-Sponsored Unified Parkinson’s Disease Rating Scale, *PIGD* postural instability gait difficulty, *iAnti* improvement in anti-saccade success rate. Patients are sorted according to iAnti score.

**Figure 1:**
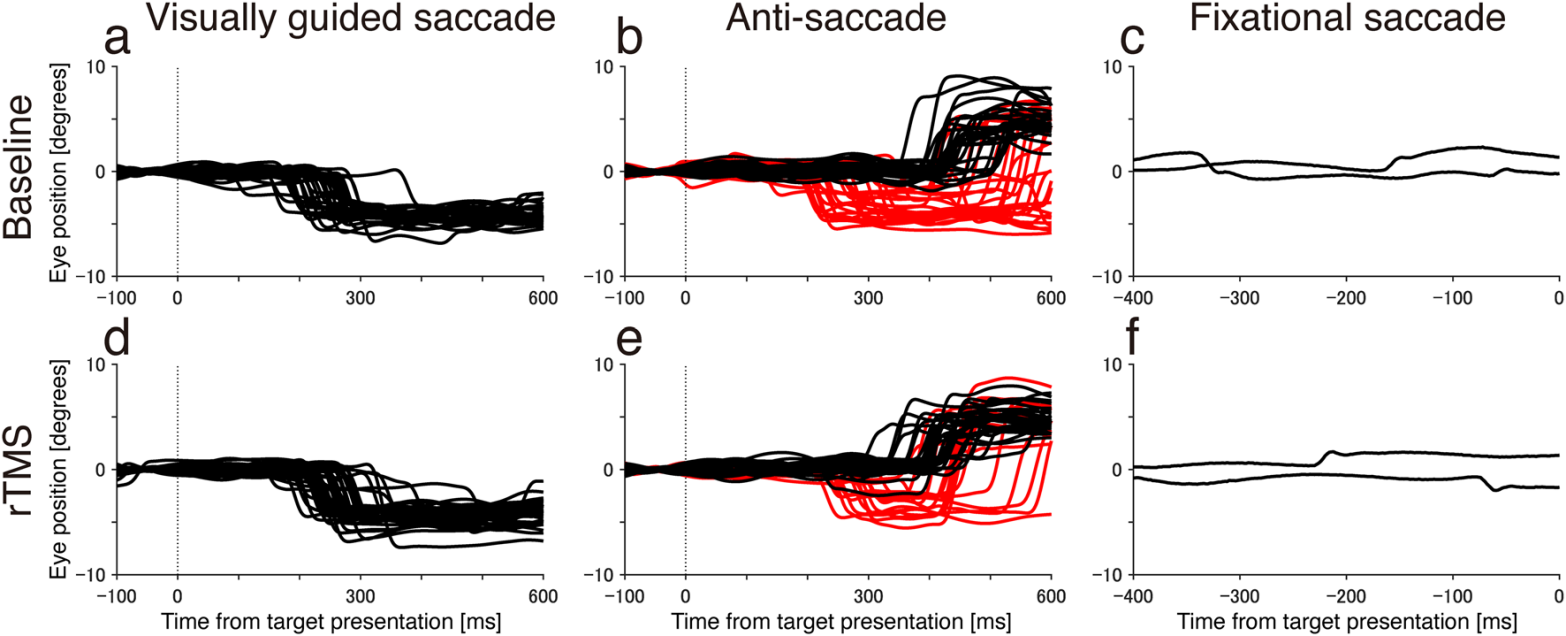
Traces of visually guided saccade, anti-saccade, and fixational saccade. Traces during baseline (top, **a**–**c**) and rTMS (bottom, **d**–**f**) conditions in a single patient are shown. Black downward deflected lines for visually guided and upward deflected lines for anti-saccade traces indicate successful saccade. Red downward deflected lines for anti-saccade traces indicate erroneous saccades toward a visual target. (**c**, **f**) Typical example traces during fixation, including small fixational saccades, are shown.

Figure 1 illustrates typical examples of visually guided saccades, anti-saccades, and fixational saccades for baseline and rTMS conditions. In anti-saccade for baseline condition, this participant made many erroneous saccades toward visual stimulus (Fig. 1b, downward red lines). While in an experiment conducted after rTMS treatment on another day, erroneous saccades were decreased (Fig. 1e). There was no change in the reaction time and gain of visually guided saccades, the reaction time of anti-saccades, and occurrence frequency of fixational saccades.

Table 2 summarises the effects of rTMS on MDS-UPDRS and saccade performance. We found that rTMS treatment significantly improved motor ability (MDS-UPDRS Part 3 score: 25.8 ± 8.8 for baseline, 16.2 ± 10.8 for rTMS; paired *t*-test, *t* = 5.79, *p* < 0.001). Regarding the MDS-UPDRS sub-scores, there were improvements in both the PIGD (1.3 ± 0.6 for baseline, 0.6 ± 0.5 for rTMS; *t* = 5.89, *p* < 0.001) and tremor scores (0.8 ± 0.6 for baseline, 0.6 ± 0.6 for rTMS; *t* = 3.59, *p* = 0.003). Contrastingly, regarding saccade performance, there was a significant post-rTMS improvement in the anti-saccade success rate (Table 2, 30.4 ± 21.5% for baseline, 43.0 ± 26.2% for rTMS; *t* =  − 2.28, *p* = 0.04), while there was no significant change in other parameters.

**Table 2:** Post-rTMS changes in the MDS-UPDRS and saccade parameters.

	Baseline	rTMS	*t*-value	*p* (paired *t*-test)
**MDS-UPDRS part 3**	25.8 ± 8.8	16.2 ± 10.8	5.79	< 0.001
PIGD score	1.3 ± 0.6	0.6 ± 0.5	5.89	< 0.001
Tremor score	0.8 ± 0.6	0.6 ± 0.6	3.59	0.003
**Visually guided saccade**				
Reaction time [ms]	204 ± 33	197 ± 24	1.53	0.15
Gain	0.56 ± 0.07	0.58 ± 0.08	− 0.82	0.43
**Anti-saccade**				
Success rate [%]	30.4 ± 21.5	43.0 ± 26.2	− 2.28	0.04
Reaction time [ms]	341 ± 107	305 ± 62	1.65	0.12
**Fixational saccade [/s]**	0.73 ± 0.41	0.68 ± 0.41	0.65	0.53

*MDS-UPDRS* Movement Disorder Society-Sponsored Unified Parkinson’s Disease Rating Scale, *PIGD* postural instability gait difficulty, *rTMS* repetitive transcranial magnetic stimulation. Statistically significant rTMS effects are underlined (*p* < 0.05).

**Figure 2:**
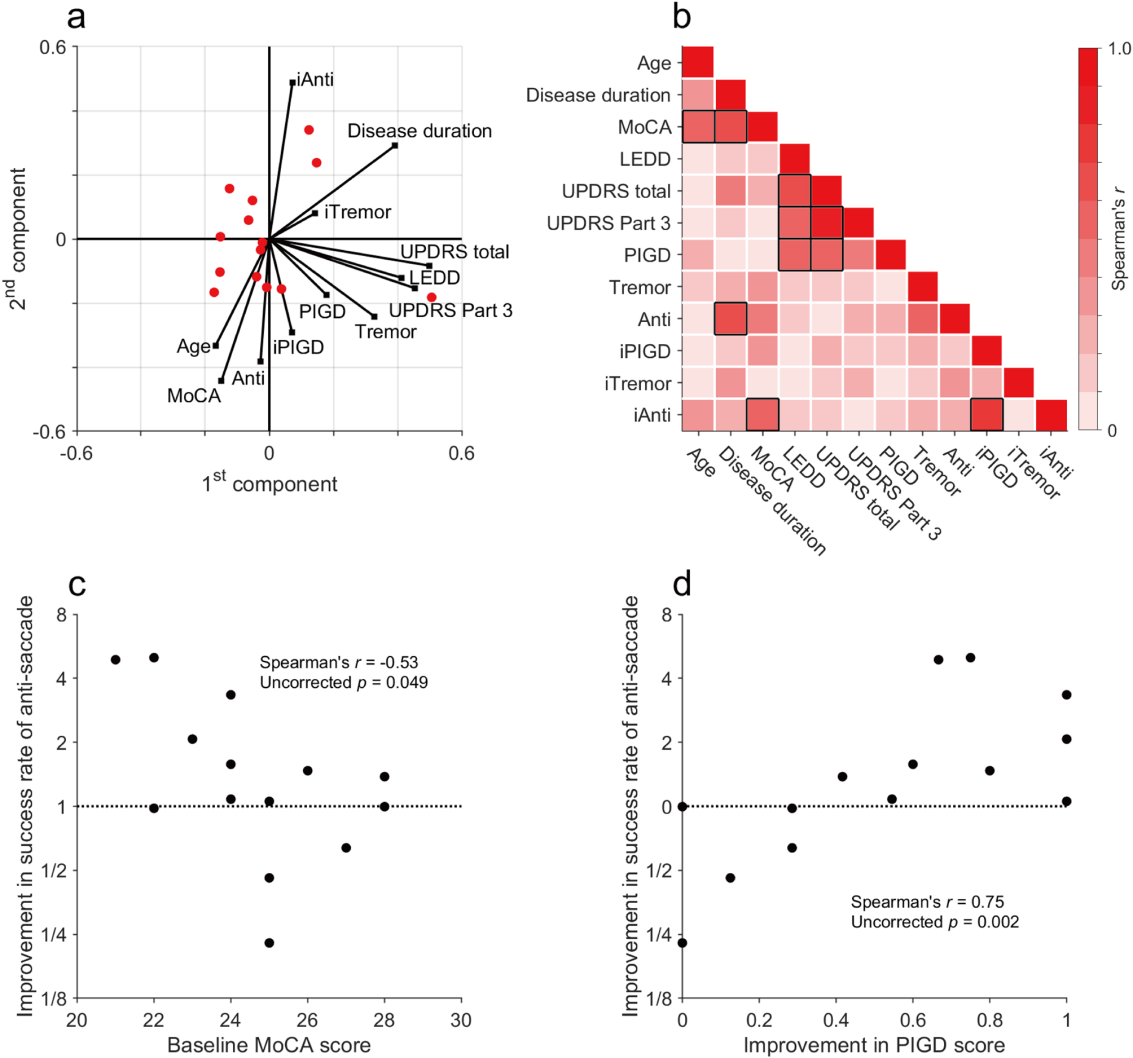
Relationships among the rTMS effects on the anti-saccade success rates, MDS-UPDRS scores, and the patients’ demographic and clinical characteristics. (**a**) We conducted PCA of the demographics as well as baseline and post-rTMS improvement in motor symptoms and saccade performance. The two main components are represented. Red dots and black lines indicate the distribution of the patients and variables, respectively. PIGD, Tremor, and Anti represent the baseline PIGD score, tremor score, and anti-saccade success rate, respectively. iPIGD, iTremor, and iAnti represent the improvement in PIGD score, tremor score, and anti-saccade success rate, respectively. (**b**) Heat maps of absolute values of Spearman’s correlations between the variables plotted in **a**. Weak correlations are in white, while strong correlations are in red. The variables surrounded by black lines indicated uncorrected *p* < 0.05. (**c**) Correlations of improvement in the anti-saccade success rate with baseline MoCA-J scores and (**d**) improvement in PIGD scores are shown.

Regarding the results of principal component analysis (PCA; Fig. 2a), the baseline MDS-UPDRS scores (MDS-UPDRS total, MDS-UPDRS Part 3, PIGD, and Tremor) and dosage (levodopa equivalent daily dose [LEDD]) formed the 1st component. Moreover, improvement in the anti-saccade success rate, baseline anti-saccade success rate, improvement in the PIGD score, baseline Japanese version of the Montreal Cognitive Assessment (MoCA-J) score, and age formed the 2nd component. Single correlation analysis revealed a considerable relationship between the improvement in the anti-saccade success rate with lower baseline MoCA-J scores (Fig. 2b,c, Spearman’s *r* =  − 0.53, uncorrected *p* = 0.049) and with improvement in the PIGD scores (Fig. 2b,d, Spearman’s *r* = 0.75, uncorrected *p* = 0.002), which did not reach significance after Bonferroni correction for multiple comparisons. There were also substantial correlations among higher baseline MDS-UPDRS total scores, Part 3 scores, PIGD scores, and higher LEDD, as well as among older age, lower MoCA-J scores, longer disease duration, and lower baseline anti-saccade success rates (Fig. 2b). Conversely, there was no such relationship between the baseline anti-saccade success rate and UPDRS indexes.

### The improvement in the anti-saccade success rate was accompanied by faster anti-saccade latency and fewer fixational saccades after rTMS treatment

**Figure 3:**
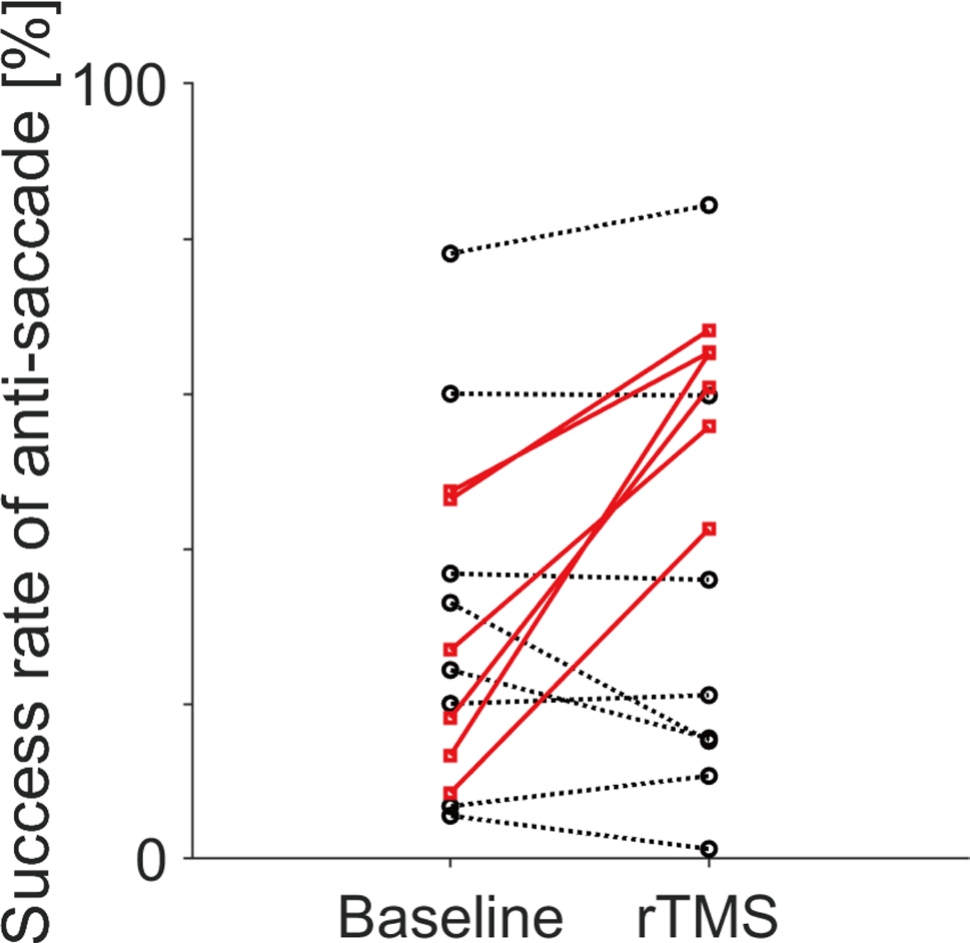
Individual changes in anti-saccade success rates. Baseline and post-rTMS performances are shown on the left and right, respectively. Lines connect data from the same individual. Solid red lines indicate significant improvement in the success rate (binomial test, *p* < 0.05) while dotted black lines indicate no significant post-rTMS improvements.

Regarding the anti-saccade success rate, an individual-level analysis revealed that, after rTMS treatment, 9 participants showed an improved trend for anti-saccade success rate (Table 1), and 6 of those showed significant improvement (Fig. 3, red lines, binomial test, *p* < 0.05). While the rest 5 participants showed a decreased trend for anti-saccade success rate, but none reached statistical significance. Thus, there were individual differences in the rTMS treatment effect on anti-saccade success rate.

Consequently, we analysed the pattern of changes in eye movement performances after rTMS treatment on participants who showed significant improvement for anti-saccade success rate (improved group, N = 6) and who did not (unchanged group, N = 8) using a generalized linear mixed-effect (GLME) model. Table 3 present the results of these GLME models. Regarding the latency of visually guided saccade, the significant negative effect of the fixation duration indicated a negative correlation of the latency with the fixation duration. Moreover, there were opposite polarities of significant effects in rTMS and an interaction between rTMS and group, which suggested between-group differences in the effects of rTMS on the visually guided saccade latency. Simply, the unchanged group showed a significantly shorter post-rTMS latency in visually guided saccades (coefficient of rTMS, exp [− 0.074] = 92.8% of baseline condition), while the improved group showed no significant rTMS effect (coefficient of rTMS & rTMS: Group, exp [− 0.074 + 0.073] = 99.9% of baseline condition).

**Table 3:** Relationships between the post-rTMS improvement in the anti-saccade success rate and other saccade parameters.

	Estimate	SE	*t*-value	*p*-value
**Latency of visually guided saccade**				
Fixation duration	− 0.009	0.002	− 5.49	4.63e−08
rTMS	− 0.074	0.004	− 16.97	6.83e−60
Group	0.059	0.073	0.80	0.42
rTMS: group	0.073	0.007	10.72	5.29e−26
**Gain of visually guided saccade**				
Fixation duration	0.001	0.004	0.18	0.85
rTMS	0.051	0.011	4.83	1.48e−06
Group	0.025	0.043	0.58	0.56
rTMS: group	− 0.086	0.017	− 5.06	4.60e−07
**Latency of anti-saccade**				
Fixation duration	− 0.003	0.002	− 1.52	0.13
rTMS	− 0.061	0.006	− 10.24	3.89e−23
Group	0.121	0.106	1.15	0.25
rTMS: group	0.016	0.009	1.83	0.07
**Frequency of fixational saccade**				
Fixation duration	− 0.036	0.034	− 1.04	0.30
rTMS	0.120	0.088	1.36	0.17
Group	0.029	0.266	0.11	0.91
rTMS: group	− 0.728	0.147	− 4.94	8.31e−07

*rTMS* repetitive transcranial magnetic stimulation. Statistically significant coefficients of GLME models are underlined (*p* < 0.05).

Regarding the gain of a visually guided saccade, the unchanged group showed a post-rTMS increase in saccade gain revealed by the significant effects in rTMS. While the improved group showed no significant rTMS effect revealed by the opposite polarities of significant effects in rTMS and the interaction between rTMS and the group. Regarding the latency of anti-saccade, both groups showed faster post-rTMS latency revealed by a significant negative effect of rTMS. Regarding the fixational saccade frequency, only in the improved group showed a post-rTMS reduction in the occurrence frequency of fixational saccade revealed by a significant negative interaction effect between rTMS and group.

Summarising these results, participants who showed an improved anti-saccade success rate after rTMS also had both a faster latency in the anti-saccade task and a lower fixational saccade frequency, while the unchanged group showed a faster latency both in the visually guided saccade and the anti-saccade task as well as a larger gain in the visually guided saccade task. Additionally, there was no significant effect of group on all eye movement performances, which indicated no differences in baseline performance between the two groups.

## Discussion

In this study, we examined the effects of bilateral high-frequency rTMS over the leg region of the M1 for the motor symptoms (tremor and PIGD scores) and eye movement performance of patients with PD. First, we confirmed a post-rTMS improvement in both tremor and PIGD scores in almost all participants (Table 2) and found a post-rTMS improvement in anti-saccade success rate in some participants (Fig. 3). Second, there were correlations of improvement in the anti-saccade success rate with PIGD score improvement and lower baseline MoCA-J scores (Fig. 2). Third, participants who showed improvement in the anti-saccade success rate also showed a faster anti-saccade latency and lower fixational saccade frequency after rTMS treatment (Table 3). The relationship between PIGD and anti-saccade improvements is consistent with the previous literature and may provide clues to elucidate the therapeutic mechanism of rTMS treatment in patients with PD.

There have been previous reports of a clinical effect of rTMS over the leg region of the M1 on tremor and PIGD symptoms^30–33^, two different clinical phenotypes of PD^3,4^. In this study, we confirmed the effect of rTMS on both types of PD motor symptoms (Table 2). Another study reported that rTMS to the supplementary motor area (SMA), just anterior to the leg region of the M1, has a clinical effect on patients with PD^38^. Although we cannot rule out the effect of a slight movement of participants, we believe that our result is mainly due to the stimulation of the leg region of the M1 but to the SMA, because we checked a TMS induced muscle twitch of contralateral leg and the definition of SMA they used is 3 cm apart from the leg motor area.

There were significant improvements in the anti-saccade success rate in some patients (Fig. 3). Moreover, participants who showed improved anti-saccade success rate after rTMS also tended to show faster anti-saccade latency and a decrease in the fixational saccade frequency (Table 3); however, there was no change in their visually-guided saccade performance. There have been several reports of patients with PD showing poor performances in anti-saccades tasks^10–14^. Successful execution of anti-saccade tasks requires inhibition of the reflexive response to the peripheral target as well as saccade initiation and execution towards the opposite side of the target, with patients with PD showing difficulties in both processes^12^. Improvement in both the success rate and latency of anti-saccade indicates that in this patient group, rTMS facilitates both the inhibition of the reflexive response as well as the initiation and execution of voluntary saccade. One study reported that performances in anti-saccade tasks for patients with PD were identical to those of healthy control in the single-task condition in which participants performed only the anti-saccade. In contrast, the performance was worsened in mixed visually guided and anti-saccade condition, and thus there was the potential deficit in set-shifting^11^. It is another essential possibility, but we could not address this issue further because we only used mixed task conditions. For fixational saccades, a previous study reported that the fixational saccade frequency reflected the dual-action preparation process; moreover, the probability of fixational saccade occurrence was greater in subjects showing poor performance than in those showing excellent performance^39^. We found that the improved group had a reduced fixational saccade frequency after rTMS treatment, which might reflect adequate anti-saccade preparation after rTMS.

Contrastingly, the remaining patients tend to show faster and more precise visually guided saccades, and faster anti-saccades after rTMS treatment (Table 3). There is a possibility that rTMS affects different neural pathways in these participants. It is possible that differences in the PD pathogeneses, including the emergence of PIGD and mild cognitive impairment, could influence the effects of rTMS. Whereas in this study, we found no difference in baseline saccade performances between improved and unchanged group (Fig. 2b, Table 3). There is a need for future studies to determine the factors that influence the effects of rTMS.

The gait/posture problems may be caused by deficits in motor control, including bradykinesia, dystonia, axial rigidity, and postural reflex disorder. At the same time, a link between the gait/posture problems and deficient cognitive control has been reported^5^. There have been previous reports of an association between anti-saccade difficulties and gait/posture problems in patients with PD^13,14^. Patients with PD showing freezing of gait have been shown to make significantly more erroneous saccades in the anti-saccade task, which suggests a specific impairment in inhibitory control^14^. Moreover, patients with PD with postural instability have slower anti-saccade latencies compared to those without postural instability; further, the latency correlated with some indices of postural instability^13^. Furthermore, there is significant overlap in the neural circuit controlling locomotion and saccade^40^. These results suggest that there are shared brain mechanisms involved in both difficulties in anti-saccade and PIGD.

Unlike simple prediction from these reports, we found no correlation between the baseline PIGD scores with baseline success rate and latency of anti-saccade. The absence of a finding may be due to our small sample size and the different way to characterise posture and gait performance. On the other hand, in this study, patients with PD who showed improvement in PIGD scores after rTMS treatment also showed improvement in the anti-saccade success rates. This suggests that rTMS may improve inhibitory control and voluntary initiation of movement by altering the brain mechanisms involved in both anti-saccade and PIGD.

Among various brain regions, basal ganglia would be a potential structure contributing therapeutic effect for locomotion and anti-saccade. DBS for patients with PD reportedly improves locomotor activity and has been established as a therapeutic option upon pharmacological treatment failure. Especially, DBS to GPi and simultaneous DBS to STN and PPN have been shown to induce improvement in anti-saccade performance^25,41^. Study in primates revealed that neurons in GP showed enhanced activity modulation during anti-saccade condition compared to those of visually guided saccade condition, and inactivation of the GP resulted in an increase in anti-saccade error^42^. The PPN is a central part of the mesencephalic locomotor region within the brainstem^43^. We have reported that some neurons in the primate PPN exhibited saccade-related activity^44–46^. Another study reported a correlation of anti-saccade latency with functional connectivity between the PPN and frontal eye field in healthy controls but not in patients with PD^13^. DBS to these structures could influence neural network activity and have a therapeutic effect on both PIGD and anti-saccade. rTMS to M1 might also affect distant neural network activity, including these areas and have a therapeutic effect plausibly through a similar mechanism for DBS.

Another line of studies reported that rTMS to patients with PD has been shown to restore cortical excitability^28^. A previous study using online TMS to produce “temporal lesion” over various cortical sites during anti-saccade preparation periods revealed the contribution of the frontal and posterior parietal regions on the successful execution of anti-saccade^47^. However, TMS over the midline area, possibly including leg region of the M1 and supplemental motor area, did not induce changes in saccade parameters. A brain imaging study on patients with PD reported a correlation of impaired anti-saccade performance with grey matter loss across bilateral visual and frontoparietal regions^14^, the regions implicated in executive functions, including anti-saccade^48^. Future study will challenge the relationship between the rTMS induced changes in cortical excitability and the effect of rTMS on eye movement and locomotor control.

There was a slight correlation of improved anti-saccade success rate with lower baseline MoCA-J scores (Fig. 2). Cognitive function in patients with PD has been shown to predict anti-saccade performance^49^. In our study, there was no correlation of the baseline anti-saccade success rate with MoCA-J score, which could be attributed to our small sample size. Although we did not assess post-rTMS changes in cognitive ability, it is possible that rTMS alleviates mild cognitive impairment and allows patients to execute anti-saccade tasks better. There have been few previous studies on the effect of M1 rTMS on non-motor symptoms in patients with PD. Our group previously reported that M1 rTMS improved sensory sensation in patients with PDs^32^; however, it did not improve depression symptoms^32^ and mood disturbances^33^.

This study has several limitations, including the small sample size, short intervention period, reproducibility of the rTMS effect for the same patients, and lack of sham stimulation and normal controls for clarifying the effects of rTMS on eye movement in patients with PD.

In conclusion, we confirmed and evaluated the effect of rTMS on the PD motor symptoms and eye movement performances, respectively. There was an association between the improvement in PIGD scores with that of the anti-saccade success rate following rTMS treatment. This result suggests that the anti-saccade success rate may be an indirect biomarker to evaluate the clinical implications of the effect of rTMS on PIGD motor symptoms. rTMS could affect common neural networks for PIGD and anti-saccade, and the detailed brain mechanisms, plausibly including the frontoparietal regions and PPN, remain to be clarified by future work.

## Methods

### Participants

We enrolled 14 patients with PD (8 females, 6 males; mean age, 69.6 ± 10.3 years) who met the United Kingdom Brain Bank criteria^50^ and were outpatients in Osaka University Hospital (Osaka, Japan). Table 1 presents the patients’ demographic characteristics. All study assessments took place in the ‘on’ state for each patient. PD symptoms were assessed using the MDS-UPDRS score^37^. We calculated the mean MDS-UPDRS tremor (11 items) and PIGD scores (5 items) based on the original classification methods^4^. Mild cognitive impairment was assessed using the MoCA-J scores^51–53^. The LEDD was calculated based on drug correspondences as per the conversion formula^54^. All patients provided written informed consent, and their anonymity was ensured.

### Ethics and dissemination

The study protocol complied with the Helsinki Declaration and was approved by the Ethics Committee of Osaka University Hospital. This clinical study was registered with the University Hospital Medical Information Network Clinical Trials Registry (Number: UMIN 000017888; Date of first registration: 10/12/2014).

### Experimental paradigm

We obtained baseline and post-rTMS measurements of motor symptoms and saccade performance on separate days. On the experimental days, the participants took their prescribed levodopa and/or dopaminergic agonist medications for symptom control.

### rTMS procedure

All the employed rTMS parameters were in accordance with previously established guideline^55^. We applied rTMS using a figure-eight coil connected to a magnetic stimulator (MagVenture, Inc., Farum, Denmark) containing a coil with an external loop diameter of 7 cm. The coil was placed over the bilateral leg region of the M1 confirmed by the TMS induced muscle twitch of contralateral leg. It was first applied over the side contralateral to the more severely affected side and subsequently, over the other side. The rTMS was applied at 90% of the resting motor threshold (RMT), defined as the minimum intensity needed to induce one visible muscle twitch, which corresponded to the RMT measured using motor-evoked potentials^56^. The stimulation frequency was 10 Hz while the stimulation duration of the pulse train was 5 s with an inter-train interval of 25 s; moreover, 10 trains were delivered over each side. All patients received 1000 pulses per session.

### Clinical evaluation

The same neurologists performed the baseline and post-rTMS clinical evaluations of the same patients. All evaluations were performed in the ‘on’ state after the same time interval between consecutive medications for each patient. To estimate the rTMS treatment effect on motor symptoms, we evaluated the post-rTMS MDS-UPDRS Part 3 and tremor- and PIGD-related Part 2 scores. Improvements in the tremor and PIGD sub-scores were estimated as follows:

$$\mathrm{Improvement} \, \mathrm{in}\, \mathrm{motor} \, \mathrm{scores}= 1- (\mathrm{after} \, \mathrm{ rTMS} \, \mathrm{ score}/\mathrm{baseline}\, \mathrm{ score})$$

Here, 0 indicates no change in motor scores, while 1 indicates no post-rTMS motor symptoms.

### Eye movement assessment

Eye movement assessments were performed immediately after the clinical evaluation. Regarding eye movement assessment, binocular eye positions were measured at temporal and spatial resolutions of 500 kHz and 0.01°, respectively, using the iView X Hi-Speed system (SensoMotoric Instruments, Teltow, Germany). In this study, the participants faced a 19-inch liquid crystal display placed at a 30-cm distance from their eyes. For stable eye recording, the participants were supported using a bite-bar, chin-rest, and forehead-rest. Because we used a stationary eye tracker, we obtained eye movement data from patients with mild to moderate PD severity^57^. Visual stimuli were presented using the Psychtoolbox in MATLAB (The Mathworks, Natick, MA, USA).

Participants performed mixed visually guided and anti-saccade paradigm tasks based on trial-by-trial task instructions^58^. Each trial began with a fixation point (size, 0.6°; colour, red; luminance, 117 cd/m^2^) being presented at the centre of the screen; then, they were required to direct their eyes towards the fixation point within 30 s. The fixation point shape (square/diamond), which was counterbalanced across participants, cued them to either saccade towards the stimulus (visually guided saccade) or the opposite direction (anti-saccade). After steady fixation for varying durations (mean: 1000 ms; range: 700–2300 ms), the fixation point disappeared with the simultaneous appearance of another peripheral target (size: 0.6°; colour: red; luminance: 117 cd/m^2^) at 5° upper-right, upper-left, lower-left, or lower-right (45° from the horizontal/vertical meridians). The participants made saccade either toward or away from the stimulus according to the task instructions. The participants performed approximately 40 trials in a single block where visually guided and anti-saccade tasks randomly interleaved. After an appropriate break of few minutes, participants performed a total of 3 blocks and thus about 120 trials per day.

### Extracted eye movement parameters

We extracted the following parameters: (1) reaction times to visually guided saccades; (2) gain of visually guided saccades relative to the target; (3) anti-saccade success rate; (4) reaction time to appropriate anti-saccades; and (5) occurrence frequency of fixational saccades during visual fixations before the successful visually guided saccades and anti-saccades. Small fixational saccades during visual fixation were extracted as described previously^39,59^. We measured the frequency of fixational saccades during the 400 ms period before the appearance of the saccade target in successfully completed visually guided saccade and anti-saccade trials. The ratio between the baseline and post-rTMS scores was used to estimate improvements in the eye movement scores. Any data affected by eye blinks and body movement were omitted from analyses.

### Statistical analysis

The paired *t*-test was used to compare the baseline and post-rTMS MDS-UPDRS scores and saccade performances (Table 2), after we confirmed that the sample distributions did not significantly differ from the characteristics of a normal distribution (The Shapiro–Wilk Test, *p* > 0.05). We performed PCA to explore the interrelationship among the effects of rTMS on the success rate of anti-saccade and MDS-UPDRS scores, and demographic and clinical characteristics (age, disease duration, MoCA-J, and LEDD), as well as to visualise similarities/differences between these variables (Fig. 2a). Moreover, we analysed the relationship between the aforementioned variables using non-parametric Spearman’s correlation coefficients (Fig. 2b–d), because some parameters were not normally distributed (e.g. age, disease duration, etc.). For individual participants, we used the binomial test to compare the baseline and post-rTMS anti-saccade success rates (Fig. 3). Based on this statistical test, we defined participants who showed significant improvement for anti-saccade success rate (improved group, p < 0.05) and who did not (unchanged group). Subsequently, we analysed differences in the effects of rTMS on eye movement performance in patients with and without improved anti-saccade success rates (Table 3). Because changes in the fixation duration across trials could affect eye movement performance, we modelled the effect of (1) the normalised fixation duration, and also (2) anti-saccade performance improvement (improved group = 1, unchanged group = 0), (3) rTMS treatment (baseline condition = 0, after rTMS treatment = 1), (4) and the effect of interaction between-group and rTMS (after rTMS treatment on improved group = 1, others = 0) on eye movement performance, while accounting for individual differences and target location across participants by constructing a GLME model^60^ using *fitglme* in the Statistics and Machine Learning Toolbox in MATLAB. We employed Poisson link functions for reaction times of visually guided saccades and anti-saccades and the fixational saccade number (nonnegative count) and normal link functions for gain (continuous) to fit the GLME.

## Data Availability

The datasets generated during the current study are available from the corresponding author on reasonable request.
